# The Role of an Endodontist in Victim Identification: A Narrative Review on Forensic Endodontics

**DOI:** 10.7759/cureus.53391

**Published:** 2024-02-01

**Authors:** Ruaa A Alamoudi

**Affiliations:** 1 Endodontics Department, Faculty of Dentistry, King Abdulaziz University, Jeddah, SAU

**Keywords:** dental soft tissue, dental hard tissue, radiographs, forensic dentistry, endodontics, dental materials, dental identification, dental anatomy

## Abstract

This narrative review highlights the role of endodontists and the significance of various dental tools in forensic dentistry. An online search was conducted in peer-reviewed journals, including MEDLINE (Ovid), PubMed, Web of Science, Scopus, and Google Scholar databases, to retrieve studies regarding “the role of an endodontist in victim identification using different tools”. The searches used controlled vocabulary and free-text terms. Articles written in English and published from 1923 to 2023 were selected. An essential stage in forensic dentistry is dental identification of the dead person and is regarded as an initial step for both judicial and humanitarian purposes if fingerprint records are missing or the remains have undergone significant changes. Endodontists should be aware of all available dental tools that aid in identification. The four fundamental tools for identification are dental radiographs, hard and soft dental structures, and dental materials. Dental radiographs provide a substantial nondestructive record for estimating age and sex. Moreover, maxillofacial hard and soft structures provide important tools for individual identification as they are considered the strongest structures in the human body and can withstand severe chemical and temperature changes. In addition, endodontic and restorative materials can be identified under different conditions and serve as excellent forensic identification measures.

## Introduction and background

Endodontics, as defined by the American Association of Endodontics (AAE), is a dental specialty focused on the structure, function, and diseases of the dental pulp and surrounding tissues [1]. This field encompasses the study and application of both fundamental and clinical sciences, covering topics such as the biology of healthy pulp, causes, diagnosis, prevention, and treatment of pulp-related diseases, as well as conditions affecting the tissues around the tooth root [2]. Forensic dentistry encompasses the examination, analysis, assessment, and presentation of dental evidence, aiming to provide scientific and unbiased information in legal proceedings. Forensic dentistry obtains sufficient information to recognize a subject based on comparing antemortem dental records such as medical and dental histories, radiographs, odontograms, cast models, etc. with postmortem records obtained from human remains, cadavers in progressed state of deterioration, or bodies that are carbonized [3]. It also helps forensic anthropologists classify human skulls, which can be crucial for identifying unknown skeletal remains [4]. A key step in forensic dentistry is the dental identification of a deceased person, which is considered fundamental for both judicial and humanitarian reasons [5]. Dental identification is important if fingerprint records are missed or the remains undergo severe changes [6]. One study reported that forensic dentistry helps identify around 60% and adds to about 30% of further identifications [7]. Forensic endodontists must possess a comprehensive understanding of several tools that can aid in the identification of individuals or provide authorities with the necessary information to establish crucial facts [3]. As an endodontist, understanding the importance of forensic dentistry and acquiring new skills in analyzing all data including dental structures, dental radiology, and dental materials, and with thorough conclusion, we can improve the service of justice. The aim of this narrative review is to demonstrate the role of endodontists in using different tools and measures that can aid in the victim identification process.

## Review

An online search was conducted in peer-reviewed journals, including MEDLINE (Ovid), PubMed, Web of Science, Scopus, and Google Scholar databases to retrieve studies regarding the role of an endodontist in victim identification using different tools. The searches used controlled vocabulary and free-text terms, as follows: (“forensic” [All Fields] OR “forensic dentistry” [All Fields] OR “victim identification” [All Fields]) AND (“endodontist” [All Fields] OR “endodontic” [All Fields] AND (“dental radiograph” [All Fields] OR “dental radiography” [All Fields] OR “Panoramic x-ray” [All Fields] OR “periapical x-ray ” [All Fields] OR “CBCT” [All Fields] AND (“hard dental tissue” [All Fields] OR “tooth” [All Fields] OR “teeth” [All Fields] OR “Maxilla ” [All Fields] OR “Mandible” [All Fields] AND (“Soft dental tissue” [All Fields] OR “lip” [All Fields] OR “dental mucosa” [All Fields] OR “paranasal sinuses” [All Fields] OR “tongue” [All Fields] AND (“dental materials” [All Fields] OR “endodontic material” [All Fields] OR “endodontic gutta percha” [All Fields] OR “endodontic sealer ” [All Fields] OR “dental restoration” [All Fields] OR “amalgam” [All Fields] OR “composite” [All Fields]).

The inclusion criteria for this review were articles from peer-reviewed journals indexed in the above-mentioned database, written in English, and published from 1923 to 2023. Thus, studies that did not meet the above inclusion criteria, such as articles not written in English or not reporting forensic endodontics were excluded. The titles and abstracts of all articles identified from the electronic searches were examined. Articles that clearly failed to meet the inclusion criteria were eliminated. Full-text copies of all remaining articles were further examined to establish whether the inclusion criteria were met. The investigator reviewed the remaining list of articles.

This review categorizes the role of endodontists in victim identification using different tools: dental radiographs, dental hard tissue structures, dental soft tissue structures, and dental materials.

The role of endodontist in forensic identification using dental radiographs 

Radiography plays a significant role in endodontics due to the inherent limitation of direct visualization of the root canals [8]. These radiographic images serve as a valuable source of antemortem data, capturing details of canal structure and any irregularities or abnormalities present [9]. It facilitates the analysis of anatomical structures for person identification, sex determination, biological age estimation, and violence or attack assessment. Radiographs collected after death are done in a way that is similar to those of antemortem images as possible, and should be affirmed by superimposition [10]. The main advantage of these radiographs is that they can be stored for an extended duration.

Both panoramic and periapical radiographs offer significant non-destructive data for estimating age and sex [11]. They are helpful in determining present, missing, or decayed teeth, assessing crown and root structure, examining pulpal anatomy, and confirming the presence of supernumerary teeth [12]. It is also helpful to identify root canal filling materials and coronal restorations [10]. However, these types only show the occluso-apical and mesiodistal dimensions of the tooth since they are two-dimensional images [13]. Crowding, rotations, and malposition make analyzing these radiographs difficult and inaccurate [14]. Thus, cone beam computed tomography (CBCT) can offer endodontists to distinguish such anatomical difficulties [15,16]. CBCT decreases the removal of the mandible since all details about teeth and body identification can be obtained from a three-dimensional scale without being disturbed [17].

Several studies reported that CBCT helps in the accurate calculation of pulp volumes [18,19]. One study advocated that the pulp cavity and hard tissue structures be studied using a new age estimation method based on CBCT images and examined the correlation between aging and age‑related changes in a tooth-pulp volume [20]. Moreover, various measurements of the lower jaw are essential for sex determination using CBCT tools such as gonial angle, bigonial breadth, bicondylar breadth, gonion-gnathion length, as well as ramus breadth and length [21]. Okkesim and Sezen Erhamza [22] reported a significant difference in mandibles among genders using CBCT images. In addition, Teixeira et al. [23] indicated that CBCT images showed that males had greater values for the length, breadth, and height measures of their maxillary sinuses, with the height of the sinus being the best indicator of sexual determination. Urooge and Patil [24] evaluated the CBCT of the maxillary sinus and reported that the efficiency of maxillary sinuses in sex identification was 74% in females and 68% in males. Moreover, a study conducted by Patel et al. [25] focused on assessing the potential of CBCT records as legal evidence for forensic analysis. The researchers specifically examined CBCT images of teeth before and after incineration to determine if the features necessary for identification were preserved. The findings revealed that even after subjecting endodontically treated teeth to heating up to 800°C, CBCT analysis could still identify sufficient features to aid in identification.

Thus, the utilization of digital radiographs and CT scans has proven to be valid methods for forensic personal identification. Nonetheless, a significant drawback of employing digital images in dentistry is the absence of standardized procedures that ensure the protection of radiographic data from manipulation and guarantee authentication [26].

The role of endodontist in forensic identification using dental hard tissue structures

Human teeth and bones of the maxillofacial region have a distinctive feature that facilitates personal identification. It is one of the strongest structures in the human body [27]. It is indestructible tissue and can survive and remain unchanged for thousands of years, even under extremely harsh conditions [5].

Human teeth exhibit a wide range of variations in root canal morphology, including increased occurrences of lateral canals, c-shaped canals, s-shaped canals, and other anatomical irregularities [28]. These diverse canal configurations are uncommon, making them exceptional features that can assist in identification. It is crucial for endodontist to be knowledgeable about these canal variations and their radiographic appearance, as they can aid in postmortem identification when compared to pre-existing dental records [29]. Moreover, the presence of supernumerary roots in permanent human dentitions is documented [30], and their prevalence can be as high as >30% in mandibular molars [31]. In addition, recent reports [32,33] consistently show a significant occurrence of middle mesial canals in mandibular molars, as well as a higher prevalence of double and three canals in anterior teeth and maxillary premolars, respectively, compared to previous findings. These new findings challenge previous assumptions and emphasize the need for endodontists to be aware of these variations in root canal anatomy during clinical practice.

Furthermore, teeth can be preserved without deterioration even after the death [34]. This is due to the fact that enamel is the strongest tissue in the human body and can remain intact in a variety of environments, including those with high temperatures, high acidity levels, salt, and humidity [35]. In addition, teeth are physiologically placed in their corresponding sockets and are covered by mucosal, fatty, epithelial, and muscular soft tissues, as well as cortical and spongy bones. These layers of tissues protect teeth if exposed to extreme conditions for an extended period [36].

It is worth mentioning that teeth behave differently under high and intense conditions. Muller et al. [37] showed complete enamel shell separation when the temperature reaches 200°C as the tooth loses its water content and collagen matrix and evokes a strong contraction, resulting in fissures, cracks, or even fractures and causes enamel to crack at the cervical line. At 400°C, the enamel starts to split from the dentin as the dentin is carbonized, and it eventually separates from the crown, causing root fracture at 800°C. This combination of transverse and longitudinal fracture leads to cement combustion and enamel disintegration at 1000 °C. Soil compounds are another condition that exerts changes in teeth. It accelerates or delays the decomposition rate, which provides signals indicating the kind and characteristics of the soil at the burial location [38]. It has been known that soil contains a high concentration of acids [39]. If the soil environment is highly acidic, buried bone structures are more prone to changes [38]. Highly acidic soils decompose bone and teeth rapidly by dissolving their hydroxyapatite inorganic minerals. Mazza et al. [35] documented changes in tooth structures over time after exposure to different acids and showed that even teeth in direct contact with acid may be assessed and recognized until the late stages of destruction. Moreover, bodies submerged in water display typical thanatological results, such as pink teeth, which may be a dental alteration linked to damp or moist settings [40].

Teeth aid in age and sex determination. Based on tooth development, dental age estimation began during fetal life and continued until young adulthood [41]. Dental age estimation in adults is based on degenerative changes in the tooth [42]. Gustafson pioneered the calculation of tooth-pulp volumes based on the amount of secondary dentin deposition and the size of the pulp space [43]. Kvaal et al. [44] created a method for estimating age indirectly depending on secondary dentin deposition from radiographs. Biuki et al. [45] revealed a strong and inverse association between pulp-to-tooth volume ratios and age. The use of posterior teeth for sex and age identification is not recommended, this is due to the changes that occurred in the pulp throughout life. Furthermore, molar teeth are more susceptible to decay, missing, filling, or even damage due to wear [46]. Maxillary canines are the teeth most frequently used for age and sex assessment because of their distinctive qualities, such as being single-rooted teeth with the biggest pulp area [47,48]. In addition, maxillary canines are less prone to decay or wear and are often kept until later in life compared to posterior teeth [47]. In a study conducted by Khalid et al. [49], the researchers examined the radiographic appearance of single-rooted teeth after obturation. They asked various clinicians to match the antemortem images with the postmortem images. The study concluded that radiographic images of single-rooted teeth possess unique characteristics that can be utilized for identification purposes.

Sex prediction is an essential step for postmortem identification [50]. Numerous studies have shown that teeth have strong sexual dimorphism. Anderson and Thompson [51] claimed that males had greater mandibular canine width and intercanine distance than females and reported a 74 % sex prediction accuracy. Another study supported Anderson and Thompson’s study and showed that the size of male and female teeth differs during measuring the mesiodistal and buccolingual dimensions of the mandibular canines, where males have bigger teeth than females [52]. Kazmi et al. [53], showed that the pulp volume of male and female canine teeth differed significantly. Additionally, there are significant differences in maxillary incisors, premolars, and first and second molars between sexes [54,55]. Moreover, tooth proportions, rather than absolute tooth size, have been proposed for sex estimation. Aitchison J [56] introduced the "incisor index" and showed males have a higher incisor index. While Rao et al. [57] proposed the "mandibular canine index" and showed it has given an accurate indication of sex.

Likewise, dental pulp tissue and the inner layer of dentin contain mitochondrial DNA that remains in the odontoblastic processes in the dentinal tubules [58]. This mitochondrial DNA from the dentinal tubules is a substantial sample in forensic conditions as it can aid in body identification that has been decomposed or burnt beyond recognition [59]. According to one study, DNA extraction from dentinal tubules can be successful after three months of death [60].

The mandible is another important structure in forensic dentistry. It has great and accurate sexual dimorphism in terms of size and form [61]. The gonial angle fluctuates during a person's life. It is obtuse at birth, then it reduces as they become older and then it rises again [62]. While females have a downward and backward rotation of the lower jaw, males have a forward rotation, which causes females to have larger gonial angle values than males [61].

The American Academy of Forensic Dentistry is concerned with identifying bite marks in criminal cases and identifying humans using antemortem dental records [63]. Bite marks are defined as semicircular injuries with two distinct arcs, one on the upper arch and one on the lower arch, with a central area that is either uninjured or has a diffuse bruise [64]. Bite marks can be seen in a variety of cases, including physical or sexual abuse, homicide, or assault [65]. Furthermore, injuries from animal bites are another possibility for victims. Animal bites can be distinguished from human bites by differences in tooth shape and arch alignment. Animal bites commonly induce shear injuries, which result in open wounds and skin lacerations [66].

The role of endodontist in forensic identification using dental soft tissue structures

Lip prints are essential soft tissues that can be utilized to determine if a suspect was present at the crime scene or not. The study of lip prints is known as cheiloscopy [67]. Lips are made up of sebaceous and sweat glands [68]. Both glands' secretions allow for the development of 'latent' lips, similar to fingerprints [69]. Lip prints of everyone are identified from the intrauterine life at the sixth week [70]. These prints then do not change. Thus, lip prints are unique for each individual and help in a person’s identification [71]. Lip print patterns are often examined during the first 24 hours following death to reduce errors caused by changes in the deceased's lip after death [72]. Additionally, lip patterns significantly differ between men and females and aid in sex determination [73].

The dorsal surface of the tongue is another soft tissue that aids in person identification. Examining the dorsal surface of the tongue's form and texture is essential because it provides numerous morphological characteristics that demonstrate its uniqueness and function as a key method of identification [74]. This can be achieved by the alginate molding impression to the lingual surface with photographic images of the dorsal surface of the tongue [75].

Palatal rugae have been shown to be an excellent tool in forensic identification. Palatal rugae are used as an alternative means of identification when other techniques of identifying a person are unsuccessful [76]. Rugoscopy is the scientific term for the study of the palatal rugae pattern [77]. After their development, palatal rugae remain unaltered throughout an individual's lifetime [78]. Caldas et al. [79] found that palatal rugae play a significant role in determining sex, as they are more prominent in males and tend to be located on the left side in both genders.

The paranasal sinus is another important tissue that remains unaffected by serious injuries to the skull [24]. According to Wanzeler et al. [80], 96.2% of men and 92.7 % of females had their sex properly identified by the sum of their maxillary, sphenoidal, and frontal sinus volumes.

The role of endodontist in forensic identification using dental materials

Endodontic obturating materials and post-endodontic restorative materials play a valuable role as a legal tool supporting the criminal demands on forensic practice. These materials have various levels of radiopacity, enabling identification through radiographic imaging [10,81]. Endodontic materials have specific compositions, and the presence of different heavy metals in these materials creates a distinct elemental fingerprint. This unique elemental fingerprint can play a significant role in aiding identification processes under different conditions [82].

One study evaluated the behavior of gutta-percha under thermal stresses, and the results demonstrated that the gutta-percha obturation material can be identified until 1100°C; however, material softening caused a "honeycomb" appearance with radiolucent areas at 600°C. When temperatures exceed 800°C, gutta-percha begins to turn a chalky whitish color, making it difficult to distinguish from incinerated dentin [83].

Additionally, the study of sealers left a unique fingerprint element because of the heavy metals they contain as provided by the manufacturer. Different sealers include various metals [84]; Bismuth may be found in EZFill (EDS, Hackensack, NJ), AH26 (Dentsply, Tulsa, OK), Grey ProRoot MTA (Dentsply, Tulsa, OK), Apexit (Ivoclar Vivadent, Amherst, NY), and Epiphany (Pentron, Orange, CA). Meanwhile, Epiphany (Pentron, Orange, CA), Tubliseal (Sybron Endo, Orange, CA), and Nogenol (GC America Inc., Alsip, IL ) all include Barium. The AH Plus sealer (Dentsply, Tulsa, OK) is the only one that uses tungsten. Silver and other elements were discovered in AH26 (Dentsply, Tulsa, OK) and Kerr EWT (Kerr, Orange, CA). Silicon is included in many root-filling products, whereas aluminum may be found in Super EBA (Harry J. Bosworth Co.) and grey ProRoot MTA (Dentsply, Tulsa, OK).

Mineral trioxide aggregate (MTA; Dentsply, Tulsa, OK) and Bioceramic (Brasseler, Savannah, GA,) are commonly used as retrograde filling materials. It is worth noting that the elemental fingerprints can be distinguished from each other due to variations in the concentrations of aluminum oxide and iron oxide [82]. Furthermore, The fingerprint of the white MTA is distinct from the fingerprint of the grey MTA due to variations in the quantities of certain metals [85].

Moreover, the elemental fingerprints of particular nickel-titanium endodontic files are another helpful tool [49]. Using scanning electron microscopy (SEM) and energy dispersive X-ray analysis (EDX), investigations have investigated the surface and elemental analysis of endodontic files under heat stress of up to 900°C. The nickel-titanium files in the root canal system were found to have a glacier-like surface coating film on them [82]. Elevated temperatures separate titanium metal from other alloys followed by surface oxidation, resulting in the formation of this layer. The identification of separated stainless-steel files is facilitated by the presence of iron, nickel, and chromium content in these files [86].

Amalgam restorative material undergoes changes in structure and superficial texture at elevated temperatures. Amalgam showed a rough occlusal surface at a temperature between 200°C and 400°C, followed by the apparition of nodules with the mercury evaporation process, in the shape of gasified bubbles when the temperature rises to 400°C up to 850°C. When the temperature is raised further, pressure forces minerals dragged by mercury to form nodules [87]. One study referred to these structures as "silver bullets" because mercury drags traces of silver, causing porosities in the amalgam surface [88]. Another study discovered a different amalgam behavior known as pigmentation. Enamel is discolored by the high-temperature dissociation of amalgam components, which causes a yellowish halo at 600°C and a golden-brown halo at 800°C [89]. The reddish-brown discoloration is also related to the oxidation maintained by copper metal brought on by the high temperatures approaching 450°C [90]. Meanwhile, pinkish pigmentation was observed on the crowns of amalgam-filled teeth when temperatures reached 1000°C and 1100°C [87].

The change in color of composite resin restoration draws attention to the fact that resin restoration plays a significant role in forensic dentistry [91]. This is related to resin restoration carbonization, incineration levels, and texture changes [92]. The resin starts the carbonization process by burning the acrylic matrix at 400°C, while it incinerates and turns chalk white at 800°C. Upon reaching the incineration point of 1000°C, the roughness gets rougher [87].

Thus, to conclude, it is highly recommended for endodontists to include the name of every restorative and endodontic material used during each procedure in the patient’s dental records. This practice ensures that accurate information is documented and can be referenced in cases where postmortem identification is required. In a case report by Berkata et al. [93], the researchers were able to establish the identity of an individual despite the absence of any teeth. This was made possible due to the presence of extruded endodontic material near the left maxillary sinus. By comparing the appearance of the extruded obturation material in the postmortem radiograph with the antemortem radiograph and records, a match was identified, confirming the individual's identity.

## Conclusions

Endodontics has become a valuable specialty in the forensic scope. The role of endodontists extends beyond performing root canal treatments and can reach establishing the identity of a deceased person. Different tools were discussed that help endodontists in identification. Radiographs provide excellent tools for identification. Understanding the dental hard and soft tissue measures is another tool that can be used to determine the victim's age and sex. Different restorative and endodontic materials that are used during root canal treatment reveal useful tools for the identification of deceased persons.
